# Evaluation of Antibiotic Residues in Raw Meat Using Different Analytical Methods

**DOI:** 10.3390/antibiotics6040034

**Published:** 2017-12-07

**Authors:** Tsepo Ramatla, Lubanza Ngoma, Modupeade Adetunji, Mulunda Mwanza

**Affiliations:** 1 Department of Animal Health, School of Agriculture, Faculty of Natural and Agricultural Sciences, Mafikeng Campus, North-West University, Private Bag X2046, Mmabatho 2735, South Africa; 21205450@nwu.ac.za (T.R.); Lubanza.Ngoma@nwu.ac.za (L.N.); ogunrinumodupe@gmail.com (M.A.); 2 Food Safety Niche Area, Faculty of Natural and Agricultural Sciences, Mafikeng Campus, North-West University, Private Bag X2046, Mmabatho 2735, South Africa

**Keywords:** antibiotic residue, ELISA, TLC, HPLC, beef, chicken, pork

## Abstract

Antibiotic residue in meat is a serious public health concern due to its harmful effects on consumer health. This study aimed at estimating the residue levels of four commonly used antibiotics in meat samples using three analytical methods (ELISA, TLC and HPLC). A total of 150 samples of raw meat from sales points were analysed for ciprofloxacin, streptomycin, tetracycline, and sulphanilamide residues. Overall, ELISA analysis showed that 56, 34, 18, and 25.3% of the samples tested positive for ciprofloxacin, streptomycin, sulphanilamide and tetracycline residues respectively while TLC and HPLC detected 21.4, 29.4, 92.5, and 14.6%, and 8.3, 41.1, 88.8, and 14.6% of the samples as containing the residues, with ciprofloxacin and sulphanilamide having the lowest and highest occurrence, respectively. Furthermore, the concentrations of antibiotic residues were in the ranges of 19.8–92.8, 26.6–489.1, 14.2–1280.8, and 42.6–355.6 μg/kg with ELISA, while HPLC detected concentration ranges of 20.7–82.1, 41.8–320.8, 65.2–952.2 and 32.8–95.6 μg/kg for sulphanilamide, tetracycline, streptomycin, and ciprofloxacin, respectively. Mean ciprofloxacin and streptomycin residue levels were above the Codex/SA MRL recommended limit, while 3% of the samples contained multidrug residues. Although some of the mean residues levels were below the permissible limits, the co-occurrence of multidrug residues in some of the samples calls for concern.

## 1. Introduction

Antibiotics are commonly used in veterinary medicine, and subsequently drug residues may persist in foods derived from animals, which may pose adverse health effects for the consumer [1,2]. Human exposure to significant levels of antibiotic residues from animal products may aggravate immunological responses in susceptible individuals and negatively affect intestinal microbiota [3]. Generally, consumption of meat (especially red meat) in South Africa was estimated to be 25.73 kg/person/year and 29.69 kg/person/year for white meat in 2007–2008 [4]. Beef, chicken, and pork are the mostly common types of meat consumed in South Africa [5].

Sulphonamides are a group of synthetic antimicrobial with broad spectrum effects against most Gram-positive and Gram-negative bacteria and protozoa [6]. Sulphonamides are widely used for therapeutic and prophylactic purposes in both humans and animals. In addition, they can also be used as additives in animal feed because prolonged ingestion of sulphonamides may have a growth-promoting effect [7,8]. Due to growing demands for meat production, several agents have been employed for animal treatment and for growth promotion. The misuse of this antibiotic can lead to antimicrobial residues [6,9,10].

Tetracycline is a broad-spectrum antibiotic used to treat a variety of infections and is also used as a growth promoter in animals. About 60% of an ingested dose of oxytetracycline is absorbed from the gastrointestinal tract and widely distributed in the body, particularly to liver, kidney, bones, and teeth [10].

The presence of antibiotics in human food is associated with several adverse public health effects, including hypersensitivity, gastrointestinal disturbance, tissue damage, and neurological disorders [11]. Tetracyclines have served for decades as a vital class of antibiotics in food animal health and production. They are used routinely in veterinary medicine for the prevention and control of disease. They are also used for growth promotion and for disease prevention in both chicken and pig production [12]. They have been widely used for the treatment of infectious diseases as well as an additive in animal foodstuffs. They are not relatively easy and inexpensive to produce, but they do not cause severe side-effects and have favourable oral bioavailability and pharmacokinetic parameters [13]. The metabolisms of tetracycline are known to bind to plasma proteins at varying degrees in different species of animals [14]. In addition, tetracycline has a short half-life (7–10 h) [15]. The acceptable maximum residues level (MRLs) for tetracyclines as recommended by the Joint Food and Agriculture Organization (FAO)/WHO Expert Committee on Food Additives are 200, 600, and 1200 μg/kg for the liver, muscles, and kidney, respectively [16].

Quinolones or fluoroquinolones (ciprofloxacin, enrofloxacin, oxolinic acid, flumequine, nalidixic acid, sarafloxacin, danofloxacin, orbifloxacin, marbofloxacin, gatofloxacin, and grepafloxacin) have been widely used in animal production and veterinary medicine for the treatment and prevention of diseases [17,18] and also in human beings [19]. Ciprofloxacin and enrofloxacin are usually prescribed for the treatment and prevention of infectious diseases in farm animals [20]. Ciprofloxacin is effective against microorganisms which are resistant to other antimicrobial agents, such as aminoglycosides, tetracyclines, macrolides, and β-lactams [21]. Enrofloxacin is known to be partially metabolized to ciprofloxacin in cattle, and ciprofloxacin achieves 25 to 35% of the concentration of the parent drug in blood [19]. Using antibiotics such ciprofloxacin for prophylaxis and the treatment of chicken and beef is authorised [17]. In the European Union (EU), countries have established a maximum residue level (MRL) of 200, 100, and 300 μg/kg for liver, muscle and kidney tissues, respectively for enrofloxacin and ciprofloxacin.

Streptomycin is an aminoglycoside produced by some *Streptomyces griseus* strains [22]. Streptomycin is well-known for its anti-tuberculosis activity, and has been used in veterinary medicine for the treatment of Gram-negative bacterial infections because of its effectiveness and low cost [23,24]. Its action is based on the inhibition of ribosomal protein that leads to death of cells [25]. Susceptible strains include *Escherichia coli*, *Salmonella* spp., *Campylobacter fetus*, *Leptospira* spp. and *Brucella* spp. [26]. Streptomycin residues can be found in agricultural products such as meat, liver, kidney, milk, and honey [26].The use of streptomycin in animal agriculture has been linked to the increased emergence of resistant strains of pathogenic bacteria that could potentially impact on human health [27,28]. For consumer protection, regulatory authorities have established residue limits for streptomycin in edible tissues and milk [28].

The analytical methods for the detection of antibiotic residues can be divided into two groups as follows: screening and confirmatory [29]. There are different screening methods for the detection of antibiotics in foods of animal origin such as thin layer chromatography (TLC) [30], enzyme-linked immunosorbent assay (ELISA) [31], the Nouws antibiotic test (NAT), a commercial ampoule test, the Premi Test [32], and others (Four-Plate Test (EU4pt). Nevertheless, several researchers, mostly from developed countries, combine imunoaffinity column, HPLC, and liquid chromatography for specificity and confirmation of results [33,34,35,36,37,38].

Several organisations such as the Food and Agriculture Organization (FAO) and the European Union (EU) have set tolerance or maximum residue limits (MRL) for antibiotic residues in food-stuffs derived from animals according to the regulations governing the maximum limits for veterinary medicine in South Africa, (Act No. 54 of 1972) and the supervision commission report of the Codex Alimentarius Commission [39]. In this study, three different analytical methods: ELISA, TLC and HPLC. were used as the method of choice because they are some of the most frequently used and cost-effective analytical methods for residues detection in foods.

To the best of our knowledge, no study has been done to determine the drug residue levels in edible tissues sold in Mafikeng. Therefore, the objective of this study was to determine the residue levels of commonly used antibiotics in chicken, pork and beef samples collected from sales points around Mafikeng, North West Province, South Africa using different analytical approaches.

## 2. Results

### 2.1. Occurrence of Antibiotic Residues in Meat Samples Using Different Analytical Methods

On the overall, ELISA analysis showed that 56, 34, 18, and 25.3% of the sample tested positive for the four antibiotic residues ciprofloxacin, streptomycin, sulphanilamide, and tetracycline residues respectively. The TLC analysis also indicated that sulphanilamide had the highest number (92.5%) of positive samples, followed by streptomycin (29.4%), ciprofloxacin (21.4%) and tetracycline (14.6%) while HPLC detected 8.3, 41.1, 88.8, and 14.6% of the samples as containing residues respectively, with ciprofloxacin and sulphanilamide having the lowest and highest occurrence (Table 1).

### 2.2. Mean Concentration of Antibiotic Residue in Meat Samples Using ELISA Method

The ciprofloxacin levels in the samples ranged from 42.6 to 355.6 μg/kg (pork muscle and liver) with overall mean concentration of 151.03 μg/kg. The maximum concentration of 355.6 μg/kg found in pork’s liver was higher than the maximum residue limits (MRL) of 300 μg/kg. Also, streptomycin residue levels in the meat samples ranged between 14.2 and 1280.8 μg/kg (chicken muscle and beef kidney) with a mean concentration of 647.09 μg/kg which is higher than the international MRL value of 600 μg/kg (muscle and liver), and the maximum concentration found in beef kidney was also above the Codex/SA MRL permissible limit of 1000 μg/kg (Table 2).

Furthermore, sulphonamide concentration ranged between 19.8 and 92.8 μg/kg with a mean value of 61.01 μg/kg, which is lower than the recommended MRL value of 100 μg/kg in the different parts of the animals, while the tetracycline level in the samples ranged from 26.6 to 489.1 μg/kg with an overall mean concentration of 168.02 μg/kg. The tetracycline residue levels in each of the samples are lower than the recommended MRL value for each product (Table 2).

### 2.3. HPLC Quality Assurance Parameters

**Figure 1:**
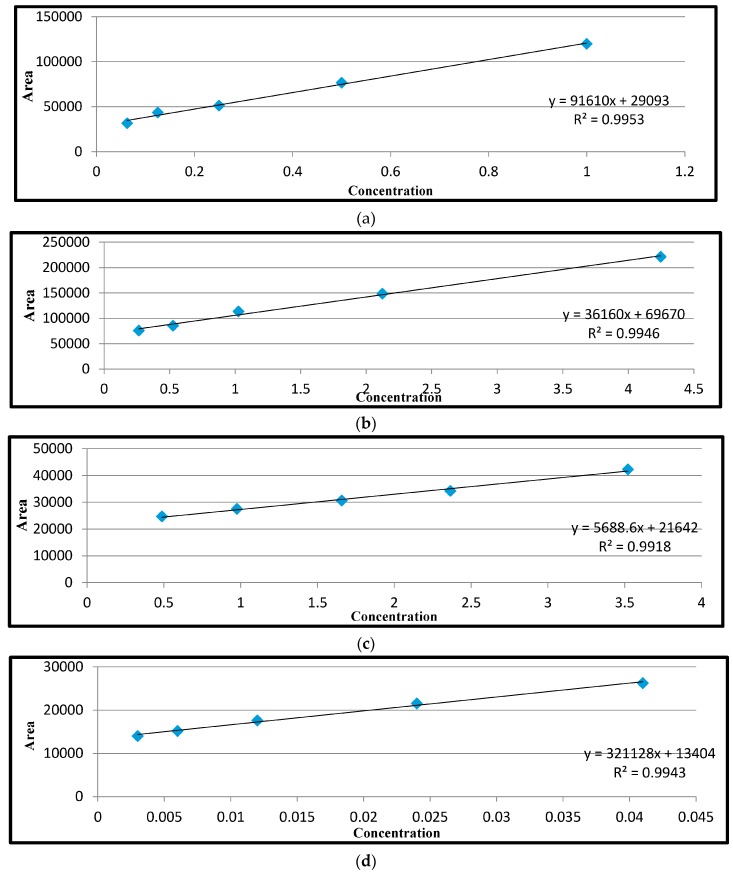
HPLC standard calibration curves for (**a**) sulphonamide standards at 1, 0.5, 0.25, 0.125, and 0.0625 μg/kg; (**b**) tetracycline standards at 0.2654, 0.5308, 1.0616, 2.1232, and 4.2464 μg/mL; (**c**) streptomycin showing linearity over the concentration range of 0.488, 0.976, 1.953, 2.485, and 3.102 μg/kg; and (**d**) ciprofloxacin standards at 0.003, 0.006, 0.012, 0.024 and 0.048 μg/kg run on HPLC under the Fluorometric Detector (FD).

The chromatographs of the reference standard and calibration curve for each of the antibiotics are shown in Figure 1. The calibration curve for sulphanilamide, tetracycline, streptomycin and ciprofloxacin showed good linearity, ranging from 0.9918 to 0.9953. Apparent mean recoveries of the residues for the spiked samples ranged between 64.33 and 80.87% (Table 3), while the limit of detection (LOD) ranged from 0.0156 to 0.122 μg/kg and limit of quantification (LOQ) ranged between 0.0488 and 4.075 μg/kg for the different antibiotic residues analysed (Table 4). The various extraction methods for the different antibiotics and other HPLC parameters are also shown in Table 4.

### 2.4. Quantitative Analysis HPLC

Analysis of meat drug resistance using the ELISA test was done for the qualitative screening of ciprofloxacin, streptomycin, sulphonamide, and tetracycline residues. The positive samples from ELISA and TLC methods were further analyzed using HPLC for quantification. The concentrations of sulphanilamide ranged between 20.7 and 82.1 μg/kg (mean; 47.9 μg/kg), of tetracycline between 41.8 and 320.8 μg/kg (mean; 88.54 μg/kg), of streptomycin between 65.2 and 952.2 μg/kg (mean; 278.75 μg/kg) and of ciprofloxacin between 32.8 and 95.6 μg/kg (mean; 66.09 μg/kg), the individual mean residues levels were below the Codex/South Africa (SA) MRL permissible limit (Table 5).

### 2.5. Multi Antibiotic Residue

Four (three livers and one kidney) out of the 150 meat samples contained multidrug residues. One of the liver samples from the supermarket had co-occurrence of streptomycin + sulphanilamide, while the other two liver samples from the butcheries had co-occurrence of streptomycin + ciprofloxacin, and tetracycline + ciprofloxacin, respectively. In addition, the kidney sample had tetracycline and sulphanilamide residue co-occurring together (Table 6).

### 2.6. Correlation between Analytical METHODS

The correlation analysis revealed a strong correlation between ELISA and HPLC methods (Table 7) for the detection of ciprofloxacin, sulphanilamide, and tetracycline in beef with *r* values of 0.71, 0.67, and 0.93 at *p* < 0.01, respectively, while no significant correlation was found between the two methods for the detection of streptomycin in beef. Furthermore, a significant correlation (*r* = 0.69 and 0.61, *p* < 0.01) was also found between the methods for the detection of sulphanilamide and tetracycline respectively in chicken product and in pork product too (*r* = 0.70 and 0.98, *p* < 0.01).

## 3. Discussion

This study affirms the potential of the ELISA method for the monitoring of selected antimicrobial residues in meat samples. Immunoassay method provided excellent sensitivity and selectivity for the determination of antibiotic residues in meat samples.

The HPLC with spectroscopic fluorometric detection (HPLC–RF) used in this study to detect the presence of tetracycline residues in the meat samples is one of the most widely used techniques for determining tetracycline residues in beef, chicken, and pork [32]. Tetracycline has a short half-life which ranges from 7 to 10 h [15]. This is because the major elimination pathway of tetracycline is through renal excretion, with about 60% of tetracycline administrated being excreted in the urine in an unchanged form [33]. In addition, the presence of sulphanilamide residue was detected using HPLC with a spectroscopic photodiode array detector (HPLC–PAD) at wavelength range of 405–495 nm as reported in previous studies [34,35,36]. However, the diode detector wavelength in this study was higher than that of previous studies [29,37] which resulted into a higher yield recovery of 71–76%. Streptomycin residue was also detected at approximately 5–7 min at a PAD wavelength range of 280–450 nm, which corresponds with methods reported in previous studies [19,38] with high yield recovery of 74–81% which also falls within the range (65–99%) reported in previous study [38].

Furthermore, ciprofloxacin was detected using HPLC with spectroscopic fluorometric detection (HPLC–RF) as described by Kirbis and co-workers [39]. There was a high yield recovery of 75–84% detected between 13.8 and 15 min (280–450 nm) as reported in previous studies [40,41]. Ciprofloxacin was firstly screened for in collected samples using the ELISA method and confirmed with TLC under ultra-violet (UV) light at 254 nm and 365 nm, which contradicts the methodology used in a previous study [42]. The detection of ciprofloxacin residues in this study might be due to the farmers not respecting the withdrawal period, or due to metabolized ciprofloxacin in the liver and its half-life range of between 2 and 6 h [43].

This study revealed that the ELISA method detected higher concentration of quinolones, streptomycin, and sulphonamides in the meat samples, which were above the MRL international standard; this might be as a result of the ELISA method analyzing the cross-reactivity of the corresponding substance in the buffer system. Furthermore, the differences in concentrations levels of ciprofloxacin and sulphonamide noted in this study between the ELISA and HPLC methods might be explained by the cross-reactivity or inability of ELISA method to discriminate between ciprofloxacin and enrofloxacin, between all antibiotics of the sulphonamide group, or between the tetracycline groups.

The correlation analysis also showed that the ELISA and HPLC methods seem to be better methods for the detection of sulphanilamide and tetracycline in all the meat products than for the detection of ciprofloxacin and streptomycin.

Despite the detection of antibiotic residues from all the organs, the liver stood out as the organ with the highest detection level (30%) and this could be as a result of the roles the liver plays in animals and humans. The liver helps in protein synthesis and also in metabolism and detoxification processes. Most of the toxic substances and residues are metabolized in the liver [43,44]. In addition, Al-Awar et al. [44] reported that acute liver injury has been described as the leading cause of drug withdrawal based on safety grounds. Similar studies conducted by Naeem et al. [45] found antibiotic residues in liver and kidney samples, which corroborate the findings of this research work.

Furthermore, Muriuki et al. [46] in Nairobi, Kenya, found tetracycline residues in animal tissues viz, 60 (24%) in liver; 35 (14%) in kidney, and 19 (7.6%) in muscle samples. In addition, Olatoye and Ehinmowo [47] in Nigeria, also found that the residue level in the liver was the highest, followed by the kidney, and lastly, muscle with 80%, 55.0%, and 28.3% respectively.

However, the findings of this study contradicted the report of Elnasri and others [48] who reported higher antibiotic residue in muscles (29%) than in the liver (28.3%), and kidney (21.4%). In contrast, Hala [49] in Khartoum found a higher residue percentage of 24.6% in kidneys. Furthermore, in Tabriz, Iran, Abbasi et al. [16] found 25.8% of tetracycline residues in muscle samples, 31.8% in liver samples, and 22.7% in kidney samples in concentrations beyond the maximum residue limits (MRLs).

The presence of antibiotic residues coupled with multidrug residues in some of the meat samples calls for concern, as this could pose serious public health risks to humans and animals, such as toxicity and resistance development.

## 4. Materials and Methods

### 4.1. Sampling Location

This study was carried out at Mafikeng, North West province, South Africa. Mafikeng is located between 25 and 28 °C south of the equator and 22 and 28 °C longitude east of the Greenwich meridian. Mafikeng city shares an international border with the Republic of Botswana in the North and 260 km West of Johannesburg. Climatic conditions vary significantly from west to east. The western region receives less than 300 mm per annum, and the central region around 550 mm p.a., while the eastern and south-eastern regions receive over 600 mm per annum [50].

#### Sample Collection and Preparation

**Figure 2:**
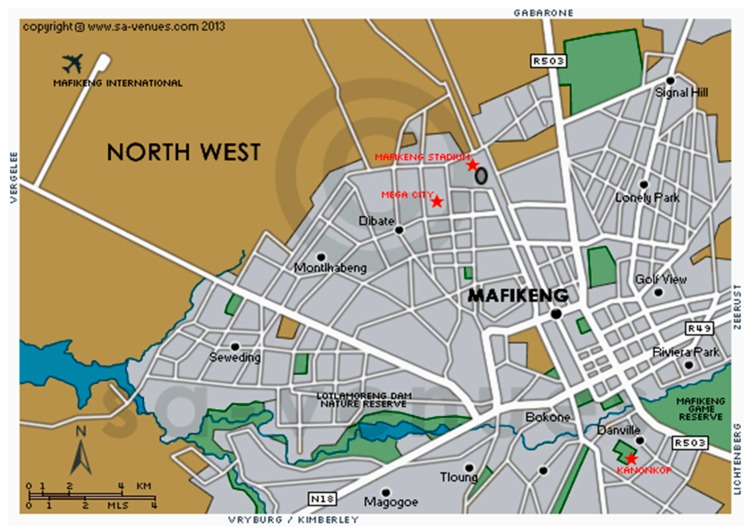
Map of sampling areas in Mafikeng (www.sa-venues.com). Accessed 02 November 2016.

A total of 150 samples (50 chicken, 50 pork, and 50 beef) of raw meat muscle, liver, or kidney was randomly collected between May and September 2014 from butcheries and supermarkets around Mafikeng Area (Figure 2). In order to ensure repeatability of the results, 10 samples were collected per month per species (Butchery and Supermarket). Collection dates and places were recorded with corresponding codes simultaneously. All samples were collected within the recommended dates for consumption. After collection, samples were packed in properly labelled sterile polyethylene bags and transported under a complete aseptic condition in an icebox to the Animal Health Laboratory of the North West University, Mafikeng Campus for extraction.

### 4.2. Chemical and Reagents

RIDASCREEN ELISA test kits (sulphonamides Art: R3004, streptomycin Art: R3103, tetracycline Art: R3505, and quinolones Art: R3113) were obtained from Biopharm AG (Darmstadt, Germany). Formaldehyde (CH_2_O), triethylamine (C_6_H_15_N), methanol (CH_3_OH), hydrochloric acid (HCl), perchloric acid (HC_l_O_4_), orthophosphoric acid (H_3_PO_4_), citric acid (C_6_H_8_O_7_), citric acid (C_3_H_4_OH), *n*-hexane (CH_3_(CH_2_)_4_CH_4_), oxalic acid (C_2_H_2_O_2_), and trichloroacetic acid (CC_l3_COOH)(TCA) were obtained from Merck (Merck, Germany). Acetonitrile (CH_3_CN) and acetone (C_3_H_6_O) were obtained from Sigma Aldrich (Sigma Chemical Co., St. Louis, MO, USA). Phosphate buffer solution (PBS) was prepared by dissolving 8.94 g sodium chloride, 0.77 g disodium hydrogen phosphate, and 0.18 g potassium dihydrogen phosphate in 1000 mL deionizer water and adjusted to the proper pH using 1 M hydrochloric acid. Distilled water and deionizer water was obtained from Animal Health Laboratory North West University. Solutions prepared for HPLC were passed throughout a 0.45-μm nylon membrane filter (Whatman, Little Chalfont, UK) prior to use. Stock solutions of sulphanilamide, streptomycin, ciprofloxacin, and tetracycline were obtained from Sigma Chemical Co., St. Louis, MO, USA. All solvents were HPLC standards.

### 4.3. Equipment

Vortex obtained from White Sci was used to mix, and solid phase extraction (HyperSep SAX C_18_ cartridge column, Waters, Milford, MA, Mass., USA). Corning^®^ LSE^TM^ digital dry bath heater (model D1200-230V, Corning^®^ LSE^TM^, New York, NY, USA) was also employed. In order to run the HPLC analysis for all samples and for all antibiotics, the following equipment was used: centrifugation was performed to separate two immiscible liquids (Z 200 A model, Herle, laboratechnic-Gmbh, Germany). HPLC was performed using the Shimadzu Class-VP Series model (Shimadzu, Kyoto, Japan) equipped with a SIL-20 auto injector (Shimadzu, Kyoto, Japan) with sample cooler and LC-20A solvent delivery units on-line vacuum degassing solvent delivery unit. The separation was done using Nucleosil C_18_ (5 μm, 150 × 4.6 mm Shimadzu, Kyoto, Japan). A fluorescence detector (RF-20 A XL) and SPD-M 20 A Deode Array Detector (DAD) (Shimadzu, Kyoto, Japan) were used, as well as a pH meter (Crison, Barcelona, Milan, Spain), a laboratory blender (Sunbeam Osterizer blender, Boca Raton, FL, USA) and different glass wares. All chemicals were of HPLC or analytical grade.

### 4.4. Extraction Procedure for ELISA

Analysis for the residue of the quinolones and tetracycline was done according to the manufacture’s guidelines The ELISA analysis was carried out according to manufacturer’s instruction (ELISA kit; r-biopharm, Darmstadt, Germany). The kit used was a competitive enzyme immunoassay for the quantitative analysis. Cross-reactivity might occur with the secondary antibody, resulting in nonspecific signal.

For the detection of tetracycline, the ELISA test can detect one substance or a group of related chemicals. In this study, the ELISA kit used for the detection of tetracycline was a competitive enzyme immunoassay for the quantitative analysis of tetracycline. According to the manufacturer’s instructions, the specificity of the ELISA test was for detection of chlotetracycline (70%), rolitetracycline (34%), demeclotetracycline (26%), oxytetracycline (13%), minocycline (3%), and doxycycline (2%). The specificity of the RIDASCREEN^®^, tetracyclin kit was determined by analysing the cross-reactivates to corresponding substances in buffer system. The limits of detection (LOD) and recovery rate of this test in meat were approximately 2 ppb and 99% respectively.

For sulphonamides, the kit used was a competitive enzyme immunoassay for the quantitative analysis of sulphamethoxypyridazine (100%), sulphapyridine (100%), sulphamethoxydiazine (75%), sulphamethaxazole (58%), sulphadimethoxine (41%), sulphadquinoxaline (34%), sulphachroropyridazine (19%), sulphamerazine (15%), sulphadoxine (10%), sulphachloropyrazine (9%), sulphaguanidine (5%), sulphapenazole, sulphamethazin, sulphisoxanzole, sulphanilamide (2%) and sulphacetamide (1%). The specificity of the RIDASCREEN^®^, sulphonamides kit was determined by analysing the cross-reactivates to corresponding substances in buffer system. The limits of detection (LOD) of this test in meat were 1.5 ppb in chicken, and 2 ppb in meat (pork and beef). The recovery rates were 70% for chicken and 115% for meat (pork and beef).

For quinolones, the kit was also a competitive enzyme immunoassay for the quantitative analysis of ciprofloxacin (100%), norfloxacin (100%), enrofloxacin (100%), marbofloxacin (100%), danofloxacin (100%), difloxacin (100%), flumequine (100%), ofloxacin (100%), sarafloxacine (43%) and oxalic acid (24%). The specificity of the RIDASCREEN^®^, quinolones kit was determined by analysing the cross-reactivates to corresponding substances in the buffer system. The limits of detection (LOD) and recovery rate of this test in meat were 10 ppb and 98%, respectively.

Moreover, the detection of streptomycin ELISA kit was a competitive enzyme immunoassay for the quantitative analysis of streptomycin (100%) and dihydrostreptomycine (140%). The specificity of the RIDASCREEN^®^, streptomycin kit was determined by analysing the cross-reactivates to corresponding substances in the buffer system. The limits of detection (LOD) and recovery rate of this test in meat were 20 ppb for meat, 25 ppb for liver, and 97%, respectively.

#### Analytical Procedures for ELISA

Analysis of all the antibiotics; quinolones and tetracycline was done according to the manufacturer’s instructions. The absorbance of the samples was read at 450 nm and the amount of antibiotic was calculated based on calibration curve. The reading was done within 30 min after adding stop solution. The absorbance was expressed as percentage (%) and calculated using the following formula:

$$\frac{\mathrm{Absorbance}\;\mathrm{standard}\;\mathrm{or}\;\left(\mathrm{sample}\right)}{\mathrm{Absorbance}\;\mathrm{zero}\;\mathrm{standard}}\times 100=\%\mathrm{Absorbance}$$

### 4.5. Extraction Procedure for TLC and HPLC

The presence of the various antibiotic residues in the meat samples were detected using methods previously reported in literature by various authors with minor modifications. Tetracycline residue was detected by adopting the methods as described by Thangadurai et al. [51] and Tajick and Shohreh [52] while ciprofloxacin, sulphanilamide, and streptomycin were detected following the methods of previous authors [19,35,36,39,53,54]. All residues were stored in the fridge until further analysis. A summary of the method employed for the detection of the antibiotics in the meat samples are shown in Table 4.

#### 4.5.1. TLC Analysis of Antibiotics

A volume of 20 μL of standard solution and the extract was pointed on silica plates using microsyringe automatic TLC spotter. The chromatographic chambers were saturated with the mobile phase for 30 min. The plates were transferred to TLC tank containing acetone/methanol (1:1) as mobile phase and the TLC plate (Silica gel on TLC Al foil, St Louis, MO, USA) was inserted into the TLC tank for the chromatographic process. After running the solvent to the end of the plates, they were removed immediately from the tank because over running can cause the spot to diffuse. The plates were dried using a drier and viewed under both short (254 nm) and long wave (365 nm) ultraviolet (UV) light (Spectroline model CM-10A Westbury, New York, NY, USA). A code “O” was drawn around any fluorescing or absorbing spots were marked with pencil and compared with standards.

#### 4.5.2. HPLC Analysis of Antibiotics

The analysis of ciprofloxacin and sulphanilamide residue were done using the fluorometric detection (HPLC–FD) method according to previous authors [41,54] respectively with some minor modifications, while tetracycline and streptomycin residue analyses were done using a photodiode-detector (PDA) [16,19], respectively with some modifications.

##### Recovery Test

In this study the recovery test for all antibiotics (ciprofloxacin, sulphanilamide, streptomycin and tetracycline) was performed in triplicate by spiking standards at three levels into different blank meat samples. About 2 g of the homogenized meat samples were spiked with 100 mL of the mixed standard. The spiked samples and blank sample without standard were then analysed by HPLC. Recovery was calculated by comparing the analysed concentrations with spiked concentrations by the formula:

$$\frac{\mathrm{Amount}\;\mathrm{of}\;\mathrm{residue}\;\mathrm{obtained}\;\mathrm{after}\;\mathrm{spiking}\;\mathrm{sample}\;}{\mathrm{Spiked}\;\mathrm{concentration}}\times 100=\%\mathrm{Recovery}$$

##### Standard Solutions

Stock standard solutions of sulphanilamide (sulphanilamide S2151000), ciprofloxacin (ciprofloxacin Y0000198), tetracycline (tetracycline hydrochloride PHR1041-500), and streptomycin (streptomycin sulfate S14000000) obtained from Sigma Chemical Co., St. Louis, MO, USA, were prepared by dissolving 10 mg of each compound in 10 mL of methanol in order to obtain a final concentration. These solutions were spiked in drug-free meat samples (samples which tested positive from ELISA) to determine recovery, accuracy, precision, and detection limit. All standards were protected from the light with aluminium foil and kept at 4 °C until used.

##### Fortification of Samples

Repeatability of the recovery assay was determined by analysis in triplicate of each of the three matrices (liver, kidney and muscle) spiked at three concentration levels. Fortified samples were allowed to stand for 20 min before analysis. The solutions were used in the preparation of the calibration curve. For ciprofloxacin and tetracyclines, the spiking concentration levels were 3, 6, and 18 μg/kg; for sulphanilamide, the levels were 10, 30 and 100 μg/kg; and for streptomycin, the levels were 4.5, 13.5 and 40.5 μg/kg. Data from these analyses was used to test linearity.

##### Stability

The stability of ciprofloxacin, tetracyclines, sulphanilamide, and streptomycin were first measured at ambient temperature for 48 h using both standard and meat samples. The stability of all four antimicrobials during the freeze–thaw procedure needed for sample analyses was further assessed at two different sample concentrations (9.20 μM and 33.6 μM). The samples were removed from the freezer and allowed to thaw at ambient temperature, then frozen again overnight. This process was repeated three times prior to the final stability determination performed in this study.

##### Method of Verification by Determining the Amount of Antimicrobial Recoveries

Recoveries were obtained by using samples which tested negative from ELISA to which antimicrobial standards in triplicates were inoculated (100 mL) with sulphanilamide, ciprofloxacin, tetracycline, and streptomycin, and recoveries obtained using the identical extraction method as for the samples and the same techniques on HPLC, as summarized in Table 6. The results obtained in each case were deducted from the ones obtained from the spiked ones in order to obtain the recovery. Quantification of antimicrobial residues in the samples was obtained and calculated from the peak heights extrapolated from the calibration curves of the standards using the following formula:

$$\frac{\mathrm{Amount}\;\mathrm{of}\;\mathrm{residue}\;\mathrm{obtained}\;\mathrm{after}\;\mathrm{spiking}\;\mathrm{sample}\;}{\mathrm{Spiked}\;\mathrm{concentration}}\times 100=\%\mathrm{Recovery}$$

Despite the fact that the recovery rate for tetracycline was 64%, the results were considered because it was the best method after several trials.

### 4.6. Statistical Analysis

The Statistical Analysis System (SAS^®^ software, 2010, Cary, NC, USA) (general linear models programme) was used to analyse data obtained for the antimicrobial residues in different species and tissues detected by ELISA and HPLC at 95% significance level (*p* ≤ 0.05). The three methods (ELISA, TLC, and HPLC) were compared using chi-square and Cramer’s V tests. One-way analysis of variance (ANOVA) and Dunnett’s T3 (as the post hoc analysis) tests were used to determine the significant difference between the antimicrobials at *p* < 0.05. Correlations between the different analytical methods and the antimicrobial residue were also analysed statistically using the SPSS software (version 16; SPSS Inc., Chicago, IL, USA) and values of *p* < 0.05 were considered correlated.

## 5. Conclusions

This research finding is reporting for the first time to the best of our knowledge the presence of antibiotic residues in raw meat samples (especially pork meat) sold at Mafikeng shops and butcheries.

The potential of ELISA for the monitoring of selected antimicrobial residues in meat samples was demonstrated. The high residue level in some of the meat samples calls for concern. Long-term exposure to doses of antibiotic residue in the body could lead to acute or chronic toxicity to the organs and the entire body. Their presences may also cause allergic reactions or produce drug-tolerant bacteria in humans after long exposure.

Hence, there is a need to respect the withdrawal periods of antimicrobials in order to reduce the level of antimicrobial residues in meat samples to a minimum and also to reinforce controls through regular sampling and analysis. Further studies on the different types of antibiotics used in poultry, piggery, and cattle farms in Mafikeng are recommended.

## Figures and Tables

**Table 1:** Occurrence of antibiotic residues in meat samples using ELISA, TLC, and HPLC methods.

Meat Type	*N*	Ciprofloxacin			Streptomycin			Sulphanilamide			Tetracycline		
		ELISA (%)	TLC (%)	HPLC (%)	ELISA (%)	TLC (%)	HPLC (%)	* ELISA (%)	** TLC (%)	** HPLC (%)	ELISA (%)	TLC (%)	HPLC (%)
Chicken													
Muscle	25	14 (56)	3 (12)	Nil	9 (36)	5 (20)	3 (12)	3 (12)	3 (12)	2 (8)	5 (20)	3 (12)	3 (12)
Liver	25	18 (72)	7 (28)	2 (11)	12 (48)	8 (32)	6 (24)	7 (28)	7 (28)	7 (28)	9 (36)	8 (32)	8 (32)
Beef													
Muscle	15	14 (93)	Nil	Nil	5 (33.3)	Nil	Nil	1 (6.6)	1 (6.6)	1 (6.6)	3 (20)	Nil	Nil
Liver	17	10 (58)	4 (23.5)	1 (10)	8 (47)	6 (35.2)	5 (29.4)	5 (29.4)	5 (29.4)	5 (29.4)	5 (29.4)	4 (23.5)	4 (23.5)
Kidney	18	7 (38)	Nil	Nil	8 (44)	3 (16.6)	3 (16.6)	5 (27)	4 (22.2)	4 (22.2)	5 (27.7)	2 (11.1)	2 (11.1)
Pork													
Muscle	16	8 (50)	Nil	Nil	3 (19)	1 (6.25)	Nil	Nil	Nil	Nil	3 (18.7)	1 (6.25)	1 (6.25)
Kidney	11	5 (45.5)	1 (9)	1 (9)	7 (64)	4 (36.3)	4 (36.3)	4 (36.4)	3 (27.7)	3 (27.7)	4 (36.3)	2 (18.1)	2 (18.1)
Liver	23	8 (34.7)	3 (13)	3 (13)	4 (17)	1 (4.3)	1 (4.3)	2 (8.7)	2 (8.6)	2 (8.6)	4 (17.3)	2 (8.69)	2 (8.69)
Total Occurrence (%)	150	84 (56)	18 (21.4)	7 (8.3)	51 (34)	15 (29.4)	22 (41.1)	27 (18)	25 (92.5)	24 (88.8)	38 (25.3)	22 (14.6)	22 (14.6)

*N* = number of meat samples, (%) = Percentage of positive samples,* ELISA—The combined sulphonamide standard from the kit was used for the ELISA analysis. ** = only positive samples from ELISA were subjected to TLC and HPLC analysis using the sulphanilamide standard.

**Table 2:** Mean concentration of antibiotic residue in meat samples using the ELISA method.

Meat Type	*n*	Ciprofloxacin (μg/kg)				Streptomycin (μg/kg)				** Sulphonamides (μg/kg)				Tetracycline (μg/kg)			
		*N* (%)	Mean ± SD	Range	* >MRL	*N* (%)	Mean ± SD	Range	* >MRL	*N* (%)	Mean ± SD	Range	* >MRL	*N* (%)	Mean ± SD	Range	* >MRL
Chicken																	
Muscle	25	14 (56)	135.8 ± 9.8	89.6–175.9	11	9 (36)	186.5 ± 19.2	98.44–452.9	Nil	3 (12)	47.5 ± 6.5	32.5–65.9	Nil	5 (20)	62.5 ± 23.1	41.2–82.1	Nil
Liver	25	18 (72)	178.6 ± 9.5	152.2–289.1	12	12 (48)	596.5 ± 374.6	368.8–986.4	9	7 (28)	73.4 ± 12.5	45.8–81.6	Nil	9 (36)	125.5 ± 22.5	42.56–286.2	Nil
Beef																	
Muscle	15	14 (93)	110.3 ± 9.4	89.6–146.1	5	5 (33)	770.6 ± 325.6	625.9–989.2	5	1 (7)	65.3 ± 0.00	–	Nil	3 (20)	48.6 ± 30.2	26.6–61.5	Nil
Liver	17	10 (59)	168.2 ± 9.9	145.2–316.5	9	8 (47)	614.5 ± 322.2	498.2–920.1	7	5 (29)	51.6 ± 14.8	19.8–87.9	Nil	5 (29)	92.3 ± 23.1	41.2–221.6	Nil
Kidney	18	7 (39)	120.6 ± 12.7	98.2–197.0	Nil	8 (44)	956.2 ± 322.1	614.2–1280.8	7	5 (20)	58.6 ± 14.8	37.6–73.9		5 (28)	192.2 ± 23.1	41.2–359.2	Nil
Pork																	
Muscle	16	8 (50)	93.6 ± 15.7	42.6–95.8	Nil	3 (19)	777.3 ± 313.1	620.3–875.8	3	–	–	–	Nil	3 (19)	58.3 ± 30.2	46.67–86.9	Nil
Kidney	11	5 (45)	102.5 ± 10.0	72.5–140.2	Nil	7 (64)	852.3 ± 322.2	14.2–1052.6	1	4 (36)	72.7 ± 16.1	52.8–92.8	Nil	4 (36)	198.3 ± 25.9	101.3–489.1	Nil
Liver	23	8 (35)	298.6 ± 16.7	220–355.6	3	4 (17)	420.5 ± 16.1	196.5–535.9	Nil	2 (9)	58.5 ± 4.9	48.2–69.9	Nil	4 (17)	98.2 ± 25.9	43.7–255.9	Nil
Total (%)	150	84	151.3 ± 11.7	42.6–355.6	40	56	646.8 ± 251.9	14.2–1280.8	32	27	53.4 ± 9.9	19.8–92.8	Nil	38	109.5 ± 25.5	26.6–489.1	Nil

*n* = number of meat type sampled, *N* = number of positive samples (% of samples that tested positive), SD = standard deviation, MRL= maximum residue level. *—no of samples with concentrations higher than the MRL, ** ELISA = The combined sulphonamide standard from the kit was used for the ELISA analysis.

**Table 3:** Recovery level of spiked organs: muscle, liver, and kidney.

Type of Sample Conc. (ng/mL)		Recovery (%)	R^2^	Type of Sample Conc. (ng/mL)		Recovery (%)	R^2^	Type of Sample Conc. (ng/mL)		Recovery (%)	R^2^	Type of Sample Conc. (ng/mL)		Recovery (%)	R^2^
Streptomycin				Ciprofloxacin				Sulphanilamide				Tetracycline			
Muscle	➢ 4.5	74	0.9918	Muscle	➢ 3	77	0.9943	Muscle	➢ 10	71	0.9953	Muscle	➢ 3	62	**0.9946**
	➢ 13.5	77			➢ 6	81			➢ 30	73			➢ 6	65	
	➢ 40.5	79			➢ 18	84			➢ 100	75			➢ 18	63	
	➢ 62.5	77			➢ 32	81			➢ 150	76			➢ 32	67	
	➢ 84.3	78			➢ 84	86			➢ 180	78			➢ 84	66	
Mean ± SD		77 ± 1.87		Mean ± SD		81.8 ± 3.42		Mean ± SD		74.6 ± 2.7		Mean ± SD		64.6 ± 2.07	
Liver	➢ 4.5	76		Liver	➢ 3	75		Liver	➢ 10	72		Liver	➢ 3	61	
	➢ 13.5	78			➢ 6	79			➢ 30	73			➢ 6	65	
	➢ 40.5	81			➢ 18	82			➢ 100	76			➢ 18	68	
	➢ 62.5	80			➢ 32	82			➢ 150	78			➢ 32	67	
	➢ 84.3	82			➢ 84	78			➢ 180	80			➢ 84	69	
Mean ± SD		79.4 ± 2.4		Mean ± SD		79.2 ± 2.95		Mean ± SD		75.8 ± 3.35		Mean ± SD		66 ± 3.16	
Kidney	➢ 4.5	77		Kidney	➢ 3	78		Kidney	➢ 10	71		Kidney	➢ 3	62	
	➢ 13.5	79			➢ 6	81			➢ 30	74			➢ 6	64	
	➢ 40.5	78			➢ 18	83			➢ 100	75			➢ 18	67	
	➢ 62.5	81			➢ 32	82			➢ 150	73			➢ 32	68	
	➢ 84.3	78			➢ 84	81			➢ 180	74			➢ 84	68	
Mean ± SD		78.6 ± 1.52		Mean ± SD		81 ± 1.87		Mean ± SD		73.4 ± 1.52				65.8 ± 2.68	

Conc. = concentration, SD = standard deviation.

**Table 4:** Detection methods of antibiotics in meat through high-performance liquid chromatography (HPLC).

Antibiotics	Sulphanilamide	Ciprofloxacin	Tetracycline	Streptomycin
Method of extraction	Solid phase extraction (SPE)	−	Solid phase extraction (SPE)	−
Sample size	10 g/15 mL	10 g/5 mL	5 g/25 mL	5 g/20 mL
Standards (μg/mL)	1, 0.5, 0.25, 0.125 and 0.0625	0.003, 0.006, 0.012, 0.024 and 0.048	0.5, 1.5, 3, 6 and 18	0.488, 0.976, 1.953, 2.485 and 3.102
Injection volume	40 μL	100 μL	40 μL	40 μL
Mobile phase	0.02 M phosphoric acid/acetonitrile (60.5/39.5)	0.02 M orthophosphoric acid, acetonitrile (85:15)	Acetonitrile and 0.01 M aqueous oxalic acid	Methanol/buffer (40/60)
HPLC detection	fluorometric	fluorometric	diode detector	diode detector
Time analysis	10 min	20 min	10 min	10 min
LOD	0.0156 μg/mL ± 0.0051	0.0015 μg/mL ± 0.0002	0.053 μg/kg ± 0.028	0.122 μg/kg ± 0.085
LOQ	0.75 μg/mL ± 0.065	0.0488 μg/mL ± 0.012	4.075 μg/kg ± 1.35	3.952 μg/kg ± 1.68

Limit of detection = LOD, Limit of quantification = LOQ.

**Table 5:** Quantification of antibiotic residues in meat samples with HPLC.

Meat Type	*N* (%)	Ciprofloxacin			Streptomycin				Sulfanilamide				Tetracycline			
		Mean ± SD (μg/kg)	Range μg/kg	>MRL	*N* (%)	Mean ± SD (μg/kg)	Range μg/kg	>MRL	*N* (%)	Mean ± SD (μg/kg)	Range μg/kg	>MRL	*N* (%)	Mean ± SD (μg/kg)	Range μg/kg	>MRL
Chicken																
Liver	2 (11)	65.2 ± 13.85	34.7–95.6	Nil	6 (50)	187.2 ± 2.9	106.5–325.1	Nil	7 (100)	32.5 ± 21.1	20.7–65.9	Nil	8	98.3 ± 27.7	46.8–220.2	Nil
Muscle	Nil	Nil	Nil	Nil	3 (33)	120.8 ± 3.3	65.2–386.4	Nil	2 (67)	45.8 ± 35.1	35.2–81.6	Nil	3	52.3 ± 22.2	44.3–65.2	Nil
Beef																
Liver	1 (10)	44.39 ± 0.00	Nil	Nil	5 (63)	201.2 ± 12.2	108.7–420.4	Nil	5 (100)	33.2 ± 25.3	23.7–71.5	Nil	4	71.6 ± 27.3	54.2–148.9	Nil
Kidney	Nil	Nil	Nil	Nil	3 (38)	711.6 ± 3.3	625.9–952.2	Nil	3 (60)	48.2 ± 26.90	26.9–82.1	Nil	2	181.3 ± 28.6	41.8–320.8	Nil
Muscle	Nil	Nil	Nil	Nil	Nil	Nil	Nil	Nil	1 (100)	62.1 ± 0.00	Nil	Nil	Nil	Nil	Nil	Nil
Pork																
Liver	2 (25)	34.2 ± 13.82	32.8–35.6	Nil	1 (25)	30.21 ± 0.00	Nil	Nil	2 (100)	58.6 ± 35.2	35.2–73.9	Nil	2	77.2 ± 44.3	68.6–80.3	Nil
Kidney	1 (20)	120.3 ± 0.00	Nil	Nil	4 (47)	421.5 ± 2.7	320.7–775.8	Nil	3 (75)	53.2 ± 27.6	27.9–77.9	Nil	2	54.7 ± 16.5	44.3–65.2	Nil
Muscle	Nil	Nil	Nil	Nil	Nil	Nil	Nil	Nil	Nil	Nil	Nil	Nil	1	84.2 ± 0.00	Nil	Nil
Total (%)	6 (7.14)	66.09 ± 13.83	32.8–95.6	Nil	22 (39.3)	278.7 ± 4.8	65.2–952.2	Nil	23 (85.2)	47.9 ± 28.5	20.7–82.1	Nil	22 (57.89)	88.5 ± 27.8	41.8–320.8	Nil

*N* = number of positive samples (% of positive samples), SD = standard deviation, MRL = maximum residue limit.

**Table 6:** Multi antibiotic residue in some meat samples.

S–ID	Organ/Area	Streptomycin	Tetracycline	Sulphanilamide	Ciprofloxacin
M46	L ^S^	+	−	+	−
M96	L ^B1^	+	−	−	+
M13	L ^B2^	−	+	−	+
M56	K ^B1^	−	+	+	−

S–ID = sample identity, L = liver, M = muscle, K = kidney, ^B1^ = butchery1, ^B2^ = butchery 2, ^S^ = supermarket.

**Table 7:** Correlation between ELISA- and HPLC-detectable concentrations of ciprofloxacin, streptomycin, sulphanilamide, and tetracycline residues in beef, chicken and pork.

Analytical Methods	ELISA A1	HPLC A1	ELISA A2	HPLC A2	ELISA A3	HPLC A3	ELISA A4	HPLC A4
Beef								
ELISA A1		0.71 *	−0.22	−0.08	0.10	−0.16	−0.08	−0.09
HPLC A1			−0.20	−0.10	0.03	−0.11	−0.06	−0.05
ELISA A2				0.40	0.02	−0.01	0.04	0.15
HPLC A2					0.04	−0.04	−0.09	−0.09
ELISA A3						0.67 *	0.07	0.15
HPLC A3							0.09	0.17
ELISA A4								0.93 *
HPLC A4								
Chicken								
ELISA A1		0.03	0.13	0.37	0.39	0.25	−0.01	0.30
HPLC A1			0.24	−0.06	0.26	0.33	0.05	0.047
ELISA A2				0.28	0.021	−0.19	0.06	0.19
HPLC A2					−0.14	−0.08	0.03	0.00
ELISA A3						0.69 *	0.22	0.56
HPLC A3							0.14	0.27
ELISA A4								0.61 *
HPLC A4								
Pork								
ELISA A1		0.04	−0.11	−0.06	−0.05	−0.08	0.01	−0.05
HPLC A1			−0.12	−0.07	−0.09	−0.07	−0.06	−0.06
ELISA A2				0.52 *	0.10	0.10	−0.09	−0.1
HPLC A2					0.11	0.34	−0.04	−0.06
ELISA A3						0.70 *	0.16	0.11
HPLC A3							−0.11	−0.11
ELISA A4								0.98 *
HPLC A4								

NS = non significant, * = Correlation is significant at 0.05 level, A1 = ciprofloxacin, A2 = streptomycin, A3 = sulfanilamide, A4 = tetracycline.
